# The Entropy of Deep Eutectic Solvent Formation

**DOI:** 10.3390/e20070524

**Published:** 2018-07-12

**Authors:** Yizhak Marcus

**Affiliations:** Institute of Chemistry, The Hebrew University of Jerusalem, Jerusalem 91904, Israel; ymarcus@vms.huji.ac.il

**Keywords:** deep eutectic solvent (DES), molecular volume, standard entropy, hydrogen bonding

## Abstract

The standard entropies *S*_298°E_ of deep eutectic solvents (DESs), which are liquid binary mixtures of a hydrogen bond acceptor component and a hydrogen bod donor one, are calculated from their molecular volumes, derived from their densities or crystal structures. These values are compared with those of the components—pro-rated according to the DES composition—to obtain the standard entropies of DES formation Δ_f_*S*. These quantities are positive, due to the increased number and kinds of hydrogen bonds present in the DESs relative to those in the components. The Δ_f_*S* values are also compared with the freezing point depressions of the DESs Δ_fus_*T/*K, but no general conclusions on their mutual relationship could be drawn.

## 1. Introduction 

Deep eutectic solvents (DESs) are a response to the search for neoteric reaction media that are “green” and readily made from inexpensive ingredients. Such solvents should conform to the requirements from “green” solvents, namely, that waste is prevented in their synthesis and processing, no toxic materials should result from the process, materials or processes that are hazardous are avoided, renewable feedstock are preferred, catalysts are preferable to stoichiometric reagents, and degradable products and reagents are preferred, as are minimal quantities of recyclable solvents.

DESs are binary mixtures that are liquid at ambient conditions and freeze (or form glasses) at temperatures considerably below those at which the ingredients do. They exhibit a network of hydrogen bonds between an acceptor (generally a salt, but not necessarily) and a donor, and are able to dissolve a great variety of solutes. The question arises as to why these eutectics have such relatively low freezing (glass formation) points. Examples of the freezing point depression of DESs relative to the composition-pro-rated values of the components are the 1:2 eutectic of choline chloride and urea, Δ_fus_*T/*K = 178 [1] and the 1:1 eutectic of lithium iodide dihydrate and water, Δ_fus_*T/*K = 116 [2]. A partial answer, at least, to this query that has been suggested is the significant impact of the many different types of H-bonds existing in the DESs that could be expected to increase the entropy of the system, and thus favor eutectic formation [3]. 

In the present study the standard entropies of DESs at 298.15 K and ambient pressure are calculated from data in the literature and compared with those of the components, pro-rated according to the DES composition, to obtain the standard entropies of formation Δ_f_*S*.

The question of whether and how the entropies of formation of the DESs affect the melting properties of the DESs is explored. 

## 2. The Data 

The standard molar entropies of a DES (subscript _E_) relative to its hydrogen bond acceptor (subscript _A_) and donor (subscript _D_) components can be estimated from their molecular volumes according to Jenkins and Glasser [4,5,6,7]. This method has been applied by some authors to the DESs [8,9] and is used in the present paper, too. The molecular volumes are obtained from the densities according to

*M/ρN*_A_ = 1.6605 × 10^−3^[(*M/*g mol^−1^)/(*ρ/*g cm^−3^)] = *v*_m_/nm^3^ (1)

where *M* is the molar mass of the DES or its component, *ρ* is its density, and *v*_m_ is its molecular (formula unit) volume. DESs are treated as ionic liquids and, following Glasser [6], the expression employed for its standard molar entropy at 298.15 K and ambient pressure, *S*_298°E_, is

*S*_298°E_/J K^−1^ mol^−1^ = 1246.5(*v*_mE_/nm^3^) + 29.5 (2)

The freezing points and the densities at 298.15 K of the DESs are taken from the author’s book [2] where they are annotated. For organic components that are solid at 298 K, the standard molar entropy is, according to Glasser and Jenkins [5],

*S*_298°_/J K^−1^ mol^−1^ = (774 ± 21)(*v*_m_/nm^3^) + (57 ± 6) (3)

whereas for liquid components the values are [5]

*S*_298°_/J K^−1^ mol^−1^ = (1133 ± 7)(*v*_m_/nm^3^) + (44 ± 2) (4)

Hence, for the components before forming the eutectic solvent the sum of the entropies is

Σ_A+D_*S*_298°_/J K^−1^ mol^−1^ = *x*_A_*S*_198°A_/J K^−1^ mol^−1^ + *x*_D_*S*_198°D_/J K^−1^ mol^−1^ (5)

The crucial quantity for the calculation of the entropy of a component of the DES is its density *ρ*, from which its molecular volume *v*_m_ is derived in the manner of Equation (2). The densities and temperatures of fusion of some of the components of the DESs, not available in the Handbook [10], are from [11,12,13,14,15,16,17]. The crystal structures of some of the components, from which the molecular volumes are calculated as *v*_m_ = *v*_uc_/*Z*, where *v*_uc_ is the unit cell volume and *Z* the number of formula units per unit cell, are from [18,19,20,21,22,23,24]. In the cases of unconventional DESs based on salt hydrates and water as components [25], the standard molar entropy of the DES is calculated using Equations (1) and (2) as for the conventional ones, but for the solid hydrates the value of *v*_mA_ is better obtained from the unit cell volumes of the crystalline salt hydrates per formula unit [26] rather than from the salt density. For the salt hydrate/water DESs the standard molar entropy of the hydrogen bond donor liquid water at 298.15 K is 69.91 J K^−1^ mol^−1^ [27]. The standard entropies of formation of the DES are then calculated by

Δ_f_*S* = *S*_298°E_ − Σ_A+D_*S*_298°E_ (6)

The freezing point depression of a DES relative to its hydrogen bond acceptor and donor components is

Δ_fus_*T/*K = *x*_A_(*T*_fusA_/K) + *x*_D_(*T*_fusD_/K) − *T*_fusE_/K (7)

Note that the difference in Equation (7) has the opposite form from that in Equation (6) in order to have positive values for the depression. 

Table 1 is a representative list of the compositions of conventional deep eutectic solvents consisting of a quaternary ammonium salt hydrogen bond acceptor with a polyol, amide, or carboxylic acid hydrogen bond donor. Also listed are the mole fractions of the hydrogen bond accepting component, *x*_A_; the temperature of fusion of the eutectic, *T*_fus_; the freezing point depression according to Equation (7), Δ_fus_*T*; the molecular volume according to Equation (2), *v*_mE_; the molar entropy, *S*_E_; the entropy sum of the ingredients according to Equation (6), Σ_A+D_*S*; and the molar entropy change on formation, Δ_f_*S*. Although DESs based on quaternary phosphonium salts with various hydrogen bond donors are an important class of conventional DESs, no density or crystal structure data for their hydrogen bond acceptor components could be found; hence, they are not included in Table 1. Table 2 is a similar list for unconventional DESs consisting of a zwitterionic or a hydroxylic acceptor and a carboxylic acid donor or DESs consisting of a salt hydrate with water.

## 3. Discussion

The standard entropies *S*_198°E_ of the DESs in Table 1 are commensurate with those obtained by other workers [8,9] on the same premise shown in Table 3. The standard entropies of formation of DESs shown in Table 1 are also in good agreement with values calculated quantum-chemically [28] and also shown in Table 3 (the negative signs on two of the values in [27] are mistakes). The entropy changes on formation of the DESs are positive in all the cases treated. They are, on the whole, larger where the hydrogen bond acceptor component is a quaternary ammonium salt (Table 1) or a salt hydrate (Table 2) than for those organic DESs where this component is neutral (Table 2). This difference cannot be attributed in the cases of the neutral acceptor components to the use of Equation (2) for the nonionic eutectic liquids rather than Equation (4) for organic liquids, the slopes with respect to *v*_m_ being commensurate. The formation of the liquid DES involves an increase in entropy that may be related to the increase in the kinds and numbers of hydrogen bonds that can be formed in the binary mixtures relative to the ingredients, even if the latter are liquid themselves. Note that for the salt hydrates at least one of the ions is a strong water structure maker, even when the counter ion is a structure breaker, so that more hydrogen bonds are formed in their concentrated aqueous solutions composing the DESs.

It is futile to try to analyze the contributions to the total entropy of the eutectic mixture in terms of the translational, rotational, and vibrational modes of the components. These modes are generally unknown for the pure components, and all that can be said about the eutectic mixture is that the translational entropy should be reduced relative to the components in view of the more extensive hydrogen bonded network characteristic of the eutectic mixture.

The data listed in Table 1 and Table 2, being representative but not comprehensive, permit qualitative conclusions only to be drawn about the freezing point depressions Δ_fus_*T* of the various kinds of DESs and the entropy changes of formation of the DESs from the components Δ_f_*S*. The latter quantities are positive in all the cases, but for the conventional DESs in Table 1, Δ_fus_*T* diminishes with increasing Δ_f_*S* values, whereas for the unconventional DESs in Table 2, it increases in this direction, more for the nonionic organic DESs than for the aqueous salt hydrate DESs, but the scatter in all the cases is appreciable.

In conclusion, the question posed in the introduction—whether the entropies of the DESs compared with those of their components affects the melting properties of the DESs—cannot be answered on the basis of the data presented here. It is doubtful if a more comprehensive collection of data would be helpful in this respect, as an important obstacle in the exploration of this direction is the lack of adequate density or crystal structure data for the solid component involved in the formation of many DESs.

## Figures and Tables

**Table 1:** Representative conventional deep eutectic solvents: their composition, *x*_A_; temperature of fusion, *T*_fus_; freezing point depression, Δ_fus_*T*; molecular volume, *v*_m_; molar entropy, *S*_E_; the entropy sum of the ingredients, Σ_A+D_*S*; and the molar entropy change on formation, Δ_f_*S*.

HB Acceptor	HB Donor	*x*_A_	*T*_fusE_/K	Δ_fus_*T*/K	*v*_m_/nm^3^	*S*_E_/J K^–1^ mol^–1^	Σ_A+D_*S/*J K^−1^ mol^−1^	Δ_f_*S/*J K^−1^ mol^−1^
Choline chloride	urea	0.333	285	178	0.3599	452	145	307
	trifluoracetamide	0.333	229	193	0.4088	507	173	334
	ethylene glycol	0.333	207	158	0.3921	488	167	321
	glycerol	0.333	233	153	0.4510	549	189	360
	glucose	0.667	288	235	0.5569	675	205	469
	phenol	0.250	253	126	0.6414	771	180	591
	o-cresol	0.250	249	123	0.6759	810	193	617
	levulinic acid	0.250	262	113	0.7188	851	192	659
	malonic acid	0.500	217	276	0.2889	371	172	199
Tetramethylammonium Cl	lactic acid	0.333	204	246	0.4217	555	154	401
Tetraethylammonium Cl	lactic acid	0.333	204		0.5179	675	187	488
Tetraethylammonium Br	ethylene glycol	0.200	249	258	0.6564	788	168	620
Tetrapropylammonium Br	ethylene glycol	0.200	250	63	0.7535	898	188	710
Tetrabutylammonium Br	levulinic acid	0.200	273	49	1.1811	1382	241	1141
	malonic acid	0.500	255	49	0.7472	960	298	662

**Table 2:** Representative nonconventional deep eutectic solvents: their composition, *x*_A_; temperature of fusion, *T*_fus_; freezing point depression, Δ_fus_*T*; molecular volume, *v*_m_; molar entropy, *S*_E_; the entropy sum of the ingredients, Σ_A+D_*S*; and the molar entropy change on formation, Δ_f_*S*.

HB Acceptor	HB Donor	*x*_A_	*T*_fusE_/K	Δ_fus_*T*/K	*v*_m_/nm^3^	*S*_E_/J K^−1^ mol^−1^	Σ_A+D_*S/*J K^−1^ mol^−1^	Δ_f_*S/*J K^−1^ mol^−1^
Betaine	glycolic acid	0.333	237	146	0.117	175	140	35
	lactic acid	0.500	298	92	0.144	208	164	44
	phenylacetic acid	0.333	266	118	0.186	261	204	57
menthol	acetic acid	0.500	265	34	0.1551	222	218	4
	lactic acid	0.333	212	108	0.1485	214	196	18
	dodecanoic acid	0.667	288	26	0.2604	354	293	61
Glucose	citric acid	0.500	283	141	0.213	295	206	89
	tartaric acid	0.500	255	178	0.188	263	186	77
Octanoic acid	dodecanoic acid	0.750	282	15	0.2916	392	274	118
CaBr_2_∙6H_2_O	water	0.695	251	48	0.2255	310	306	4
Ca(ClO_4_)_2_∙6H_2_O	water	0.548	198	117	0.4654	609	322	287
KF∙2H_2_O	water	0.242	233	50	0.2587	351	91	260
KOH∙H_2_O	water	0.488	208	127	0.1270	187	93	94
LiNO_3_∙3H_2_O	water	0.171	250	76	0.3170	424	96	328
LiI∙2H_2_O	water	0.670	204	116	0.1692	240	146	94
MgBr_2_∙6H_2_O	water	0.472	230	121	0.4112	542	224	318
Mg(ClO_4_)_2_∙6H_2_O	water	0.582	204	159	0.4937	644	332	312

**Table 3:** Some deep eutectic solvents reported in the literature: their composition, *x*_A_; molecular volume, *v*_m_; molar entropy, *S*_E298°_; or molar entropy of formation, Δ_f_*S*.

HB Acceptor	HB Donor	*x*_A_	*v*_m_/nm^3^	*S*_E298°_/J K^−1^ mol^−1^	Δ_f_*S/*J K^−1^ mol^−1^
Choline chloride	urea	0.333			301 [27]
	ethylene glycol	0.333			333 [27]
	malonic acid	0.500			160 [27]
	propanoic acid	0.333	0.4445	584 [9]	
	chloroacetic acid	0.333	0.4269	562 [9]	
	trichloroacetic acid	0.333	0.5302	690 [9]	
	*p-*toluenesulfonic acid	0.333	0.6469	836 [9]	
Tetrabutylammonium Cl	ethylene glycol	0.333	0.6751	871 [8]	
	polyethylene glycol	0.333	1.6619	2101 [8]	
	propanoic acid	0.333	0.7327	942 [8]	
	phenylacetic acid	0.333	0.8785	1124 [8]	
